# What is to be Done? Why Reward is Difficult to Do Without

**DOI:** 10.3389/fpsyg.2012.00412

**Published:** 2012-10-22

**Authors:** David Spurrett

**Affiliations:** ^1^Philosophy, University of KwaZulu-NatalDurban, South Africa

Clark's synthesis of much recent work on sensory and motor systems in the brain is at once radical and curiously traditional. It is radical, among other things, concerning what representations are, how they are constructed, and what sensory and motor representations have in common. But it is traditionally cognitivist in viewing the main task of brains as being that of representing the world.

What this traditional orientation tends to neglect is the role of the brain as a system for selecting among available actions. This phenomenon has an ultimate aspect regarding the external standards relevant to assessing actions. Various behavioral ecological schemes for ranking actions in terms of their contribution to quantities such as fitness, and economic models of revealed preference, are the leading theoretical players here. The phenomenon also has a proximal aspect, which concerns the specific biological mechanisms, including neural ones, by means of which the values of different available actions might be represented, and selections between them made. On this topic the recent explosion of neuroeconomic research on decision processes in the brain is urgently relevant.

Natural agents have limited means of action, and those means have alternative – sometimes mutually exclusive – uses. That is to say the *predicament* of natural agents is fundamentally an economic one, even if it is not necessary that selection converge on a system for responding to the predicament in which economic variables are explicitly represented. Furthermore there is considerable evidence from behavioral ecology and other fields that many vertebrate behaviors in natural settings are economically efficient.

Neither the ultimate nor the proximal aspects of the problem of selecting between behaviors play a significant role in Clark's account. Natural selection, fitness, and biological descendents are not mentioned at all, and cognate concepts like adaptiveness feature in diluted form. There's similarly little mention of decision and choice as theoretically understood in economics including neuroeconomics, none of incentives, and reward and utility appear only in the course of musing over whether it is possible that cognitive neuroscience could do without reference to either (section 5.1). Clark does make some important points about action-centric *representations*, but even here does not consider the problem of action *selection*.

Of course, no survey can cover anything that anyone thinks is relevant, and it's very easy to complain about things that are left out. Clark's lack of engagement with neuroeconomics means missing a specific opportunity to make his general case even more compelling, because what is emerging in that field complements his case about sensory and motor systems in deep ways.

In his section (3.2) Clark apparently takes seriously the concern that an agent with the sort of brain that he's been describing would be expected to “seek a nice dark room and stay in it”. Clark disposes of the worry by pointing out that creatures with real biological needs should “expect” to follow exploratory strategies, and that these expectations themselves should recruit both perception and action. This is part of a reasonable and interesting response, but action selection under those conditions (as with most others) would still require some way of dealing with specific questions, such as where and how to forage, and how to trade off foraging with other expected behaviors such as predator avoidance and reproduction.

A related move appears later, in section (5.1) when he considers an austere vision of cognition that does without reference to goals and rewards, in favor of comprehensive analysis in terms of expectations. Clark correctly holds back from endorsing this possibility, but for relatively generic reasons to the effect that even if some description is in principle replaceable, it may be convenient to continue using it. This misses the main chance. Recent work on the neural implementation of decision in various vertebrates including humans has produced a body of results highly congenial to the unifying vision Clark supports.

Consider saccadic movements in rhesus monkeys. A key component in the neural implementation of these movements is the lateral intraparietal area (LIP), which comprises a topographic map integrating locations in the visual field and aspects of the muscular plans that would effect the centering of gaze on those locations. It, along with a network of other maps with varying topographies in the frontal eye fields, superior colliculus and related areas, provides a striking illustration of what Clark calls an “action-centric” representation. In addition, as studies including Platt and Glimcher (1999) and Dorris and Glimcher (2004) have shown, some activity in LIP neurons of rhesus monkeys on *visually identical* trials varies in precise ways with the relative expected rewards (or relative subjective value) from saccades to the represented location. These representations are not merely “action-centric” insofar as they combine answers to the questions “where is it?” with “how do I gaze at it?” They also include identifiable activity corresponding to the answer to “what's it probably worth for me to look at it?”

There's more. The expected relative reward values attached to saccadic and other movements are not *sui generis*. They are predictions, and ones that get updated in the light of ongoing experience. Among the key findings on this topic is that dopamine neurons do not – as previously supposed – directly encode hedonic value (because if they did they would respond in the same to expected and unexpected rewards of equivalent hedonic worth). Rather it turns out that they encode some aspects of the *difference* between experienced and expected reward (Montague et al., 1997, see also Bayer and Glimcher, 2005). While many details about the operation of this system, and its interaction with other neural systems, have yet to be determined, it is nonetheless clear that crucial features of the neural systems for attaching values to sensory events and actions operate by means of prediction error. In this respect they suggest a way of expanding the scope of Clark's claim about the importance of minimizing prediction error as a general goal of neural systems.
